# Development of behaviour change communication strategy for a vaccination-linked malaria control tool in southern Tanzania

**DOI:** 10.1186/1475-2875-7-191

**Published:** 2008-09-29

**Authors:** Adiel K Mushi, Joanna Schellenberg, Mwifadhi Mrisho, Fatuma Manzi, Conrad Mbuya, Haji Mponda, Hassan Mshinda, Marcel Tanner, Pedro Alonso, Robert Pool, David Schellenberg

**Affiliations:** 1 National Institute for Medical Research-Amani Centre, P.O. Box 81, Muheza Tanzania; 2 Ifakara Health Institute, P.O. Box 78373, Dar es Salaam, Tanzania; 3 London School of Hygiene and Tropical Medicine, London, UK; 4 Swiss Tropical Institute, Basle, Switzerland; 5 Barcelona Centre for International Health Research (CRESIB), Institut d'Investigacions Biomèdiques August Pi i Sunyer (IDIBAPS), Barcelona, Spain

## Abstract

**Background:**

Intermittent preventive treatment of malaria in infants (IPTi) using sulphadoxine-pyrimethamine and linked to the expanded programme on immunization (EPI) is a promising strategy for malaria control in young children. As evidence grows on the efficacy of IPTi as public health strategy, information is needed so that this novel control tool can be put into practice promptly, once a policy recommendation is made to implement it. This paper describes the development of a behaviour change communication strategy to support implementation of IPTi by the routine health services in southern Tanzania, in the context of a five-year research programme evaluating the community effectiveness of IPTi.

**Methods:**

Mixed methods including a rapid qualitative assessment and quantitative health facility survey were used to investigate communities' and providers' knowledge and practices relating to malaria, EPI, sulphadoxine-pyrimethamine and existing health posters. Results were applied to develop an appropriate behaviour change communication strategy for IPTi involving personal communication between mothers and health staff, supported by a brand name and two posters.

**Results:**

Malaria in young children was considered to be a nuisance because it causes sleepless nights. Vaccination services were well accepted and their use was considered the mother's responsibility. Babies were generally taken for vaccination despite complaints about fevers and swellings after the injections. Sulphadoxine-pyrimethamine was widely used for malaria treatment and intermittent preventive treatment of malaria in pregnancy, despite widespread rumours of adverse reactions based on hearsay and newspaper reports. Almost all health providers said that they or their spouse were ready to take SP in pregnancy (96%, 223/242). A brand name, key messages and images were developed and pre-tested as behaviour change communication materials. The posters contained public health messages, which explained the intervention itself, how and when children receive it and safety issues. Implementation of IPTi started in January 2005 and evaluation is ongoing.

**Conclusion:**

Behaviour Change Communication (BCC) strategies for health interventions must be both culturally appropriate and technically sound. A mixed methods approach can facilitate an interactive process among relevant actors to develop a BCC strategy.

## Background

Malaria continues to be a leading cause of pain, death and poverty in sub-Saharan Africa. Efforts have been directed at the prevention of malaria in pregnant women and young children who carry the greatest burden of disease. In addition to the promotion of treated mosquito nets, sulphadoxine-pyrimethamine (SP) is recommended by the WHO for intermittent preventive treatment of malaria in pregnancy (IPTp) in endemic countries. Intermittent preventive treatment of malaria in infants (IPTi) using SP has been shown to be a promising approach for malaria control in young children, with a protective efficacy of 20–59% against malaria in children during their first year of life [1-3]. IPTi consists of a full dose of antimalarial treatment delivered to young children at defined intervals alongside routine health contacts, such as vaccinations at the 2^nd^, 3^rd ^and 9^th ^month of life. Linking malaria control to Expanded Programme on Immunization (EPI) contacts in this way builds on the success of this well-established program which was launched in 1974 [4,5]. Further studies are underway across Africa under the umbrella of the IPTi consortium to generate evidence to inform a policy decision for public health use of IPTi [6]. A recent review by an expert committee of the US Institute of Medicine concluded that IPTi using SP decreases the incidence of clinical episodes of malaria by 20–30% [7,8],

SP was introduced as Tanzania's first-line treatment for malaria in 2001. However, adverse publicity caused widespread alarm about the safety of this drug, with publication in the national press of dramatic photographs showing people with rare, but very severe adverse events. There was concern that acceptance of IPTi using SP might be compromised. The work described here is part of a community effectiveness study of IPTi using SP in southern Tanzania. The work investigated providers' and communities' understanding of, attitudes and practices related to SP for malaria treatment and IPTp, as well as the acceptability of adding IPTi into existing immunization services. This paper reports a mixed methods approach to inform the development of a behaviour change communication strategy for malaria control with IPTi in southern Tanzania.

## Methods

The study involved work at community and health facility levels between August and November 2004, in five districts (Tandahimba, Newala, Lindi Rural, Ruangwa and Nachingwea) of Lindi and Mtwara regions, southern Tanzania. Qualitative data was collected at both levels through focus group discussions and in-depth interviews while quantitative data was collected from health providers through a modular questionnaire during a health facility survey. The districts, with a total population of 890,000 and 136 health facilities, are inhabited by a variety of ethnic groups, predominantly Makonde and Mwera, but including other groups such as Yao, Malaba and Makua. Lindi and Mtwara regions have the highest under-five mortality rates in Tanzania [9]. Geographically, the five districts include the coastal belt along the Indian Ocean to the Makonde plateau in the hinterland, extended woodlands with wild animals and an international border with Mozambique. Swahili, the national language of Tanzania, is widely spoken in the area and was used throughout data collection and in the development of the BCC materials for IPTi.

### Rapid qualitative study

Fifty-two focus group discussions (FGDs) and eight unstructured open ended interviews were held with community members and health workers between September 2004 and January 2005. Data was collected by a team of seven experienced field assistants led by a social scientist. The assistants were trained for five days on interview skills, data recording with MP3 recorders and preparation of daily summaries. Training included group work, role-play and practical fieldwork. Data was collected in ten villages, purposefully selected to represent areas with both low and high EPI coverage rates according to a household survey conducted during 2004. Selection also considered varied geographic locations including coastal areas, the Makonde plateau, semi urban and rural communities, those close to and far from referral hospitals and the international border to Mozambique.

Village leaders were briefed one day before the FGDs and asked to help prepare respondents and venues. On days of data collection, four FGDs were held, one each with "community own resource persons" (widely referred to in Tanzania by the acronym CORPs, they are people who do voluntary work in their community relating to health, agricultural extension work, water, etc.), mothers of babies and pregnant women at Reproductive and Child Health clinics, mothers of babies and pregnant women at village centres and mothers of babies and pregnant women in outlying hamlets. Three pairs of interviewers each held one FGD in the morning and took turns to conduct the fourth session in the afternoon. The FGDs and interviews were facilitated using a guide with questions on perceptions of and experiences relating to vaccination services, malaria, and use of SP, willingness to accept and views on how to implement IPTi. Community understanding of the existing health posters was assessed to determine their clarity to target audiences, in terms of text, images and take home messages. All discussions were recorded using an MP3 voice-recorder and transcribed verbatim. Debriefing notes from each FGD were prepared daily and these together with a review of transcribed text enriched the content analysis process, which involved coding of related text under corresponding themes.

### Health facility survey

A modular questionnaire was administered to health workers at all 136 government, NGO and private health facilities (including hospitals, health centres, and dispensaries) in the five districts. As well as information on the structure and function of the health system, one module of the questionnaire included questions to evaluate the health worker's perceptions of SP for treatment and prevention of malaria. This module was designed with input from an initial in-depth interview with a Public Health Nurse at a health centre in Mtwara municipality. A team of 18 research assistants plus a field co-ordinator administered the health facility questionnaire, following four days of training and pilot testing. The supervisor accompanied at least one interview each day and any discrepancies were discussed with the interviewer and later among all team members, and appropriate action taken. Questionnaires were processed using a double data entry system in DMSys software (SigmaSoft International, Chicago, IL, USA ). Data were checked for logical consistencies, completeness and quality and then summarised according to a pre-defined analytical plan using Stata 8.2 (Stata Corp LP, College Station, Texas, USA).

### Development of the BCC strategy

The BCC strategy was developed in such a way as to be feasible and low cost on a national scale. The core of the strategy was interpersonal communication between health workers and mothers at the time of giving babies EPI vaccines. This core was supported by a brand name and posters. To ensure that the content of BCC materials was in harmony with national policies and standards, an extensive series of consultations was done with national stakeholders including senior managers and staff of the Ministry of Health, the EPI programme, the Reproductive and Child Health department, the Health Education Unit, the National Malaria Control Programme and the National Kiswahili Council in Tanzania.

### Development of BCC materials

During the rapid qualitative study, results of earlier IPTi efficacy trials in other parts of Tanzania were presented to respondents before discussions about willingness to accept IPTi. The respondents who included health workers, mothers and community-own resource persons were then asked to suggest a brand name for IPTi, images, captions and appropriate channels for BCC messages.

## Results

### Perceptions of malaria in young children

Malaria was mentioned as a common childhood illness in all FGDs, with fever and crying at night as the main symptoms, occurring particularly during the heavy rains between October and April/May. This season was associated with high mosquito numbers, thought to be directly linked to malaria. Malaria was described as a nuisance to children and a trouble to their parents, particularly mothers because it leads to sleepless nights. Signs of severe malaria and anaemia were attributed to bad spirits, witchcraft, lack of medicines at health facilities and apathy *(uzembe) *among the parents. Severely ill infants were often reported to be treated at home or by traditional healers, despite the knowledge that this could risk their lives due to delays in seeking prompt care at health facilities.

*"You go for the first and second time [to the health facility] without getting medicines, and then you can't see the importance of going again when the same child falls sick. I would rather go to traditional healers because you can't get drugs at the health facility" *(FGD with mothers in an outlying hamlet).

Child deaths that occurred at home due to delayed treatment or during treatment by traditional healers were accepted because children also die at health facilities.

### Perceptions and use of vaccination services

Most respondents felt that it was compulsory for children to attend EPI clinic for vaccinations and weight measurement. Although vaccine-preventable diseases such as pertussis, diphtheria, tetanus and polio were occasionally mentioned, female respondents generally distinguished the vaccines according to how they are administered e.g. as oral drops, into the thigh or into the shoulder. Generally very few, especially among the male respondents, knew the age at which these vaccines were given. Most respondents were not aware what the vaccines protected against, except for measles vaccine, which they said was given at the 9^th ^month of age. In all areas, mothers were responsible for decision making and action pertaining to vaccination clinics. Nevertheless if the mother is sick, fathers, aunts, sisters, and grandmothers could make decisions or take a baby to clinic. The mother's role of taking a baby to clinic was justified because she can soothe the baby after vaccination by breastfeeding as well as the normal mother-child bond, expressed as the mother knowing the pain of bearing a child *(uchungu wa mwana ajuae ni mama)*. It was also generally agreed that mothers would take their babies to clinic regardless of the father's attitude, due to perceived benefits of vaccines in averting disease and mothers being responsible for caring for sick children.

*"When a child falls sick, I will be the one to suffer with the child, while my partner will be with other women" *(FGD with mothers in outlying hamlet)

*"What if I don't take this baby and then she falls sick? I will take her [to vaccination clinic] and tell him [my husband] that it is up to you, if you want to divorce me because of taking a child to clinic, we will see what happens" *(FGD with mothers, village centre)

Mothers also felt obliged to take their babies to clinic because if they did not, they might suffer certain consequences such as denial of treatment in case of illness. Health facility workers often demand the child's health card before treatment partly as a way of confirming the age and weight of a baby, and these records are marked during attendance at clinic for vaccinations and or growth monitoring.

*"We take [health cards] to [vaccination] clinic so that a child can get drugs when he or she is sick" *(FGD with mothers, village centre)

Mothers who did not use EPI were reported to exist in most areas. Reasons for non-attendance were apathy, distance to the clinic, unfriendly staff, rains, and farming, travelling away from home and fear of wild animals on the way. Some mothers were said not to take their babies to vaccination clinic because they were afraid of abscesses or fevers following injectable vaccinations and the occasional unavailability of vaccines:

*"Some mothers fear that when their babies receive an injection they will get fevers and abscesses" *(FGD, community own resource persons)

*"For example, you may be asked to take your baby to clinic to get the vaccine, but whenever you go, you might miss that vaccination even for a whole year because it is unavailable, contrary to the plans" *(FGD, mothers, village centre)

Fear of treatment denial due to lack of up-to-date vaccination records was so strong that a few areas it was reported that some mothers might write vaccination dates on their baby's health cards without actually taking them to clinic. Nearly all Community Own Resource Persons at one coastal village without a functioning health facility said they had heard of mothers in their village who wrote false dates of clinic attendance on their children's health cards so as to pretend that they had brought their children to clinic.

### Acceptance of SP in the community and among health workers

Antimalarials mentioned in FGDs included quinine, generic SP and two SP brands of Fansidar and Metakelfin. At community level, SP was particularly familiar to pregnant women who had used it for IPTp. There were mixed opinions about the benefits and side effects of SP. Many respondents had used SP safely either for themselves or for their children. However, some participants at a semi-urban village preferred Fansidar because they thought it contained less sulphur, which they associated with adverse reactions. Some women did not know whether they had received IPTp, despite saying they had received three white tablets which other participants thought were SP. These white tablets were said to be for making the mother and unborn child healthy, particularly to protect from malaria. Several respondents had heard rumours about adverse effects of SP and some had experienced dizziness (mentioned in many places), and less commonly mouth sores, fatigue, fever, rash, and miscarriage.

*"After taking SP (for IPTp) when I was pregnant, my period started"*. (FGD, Women, Semi-urban)

There was a concern that IPTp might lead to large babies, which would lead to a difficult delivery:

*"Some mothers are also scared that they may be harmed during delivery due to the large size of the unborn baby. Others decide to throw these drugs away, because an enlarged baby may cause a rupture." *(FGD, mothers, outlying area)

Occasionally, the fears were said to have led mothers to discard the SP. Although most female respondents said they had used SP for IPTp, they were aware of others who threw away SP given to them at clinic for consumption at home. One male participant during FGD with Community Own Resource Persons (semi urban) said that his wife had thrown away SP tablets which she received at clinic for IPTp, because they both suspected that the drugs might be harmful.

Hearsay was the main source of information about the adverse effects of SP while a few knowledgeable participants also recalled statements made by "experts" in the newspapers (Community Own Resource Person).

In the health facility survey, almost all health providers said that they or their spouse were ready to take SP in pregnancy (96%, 233/242) and to treat a relative with SP (95%, 231/242). About three-quarters said they had used SP to treat their own child during his/her most recent illness (72%, 132/183). Only one-fifth of health workers said they had experienced pregnant women who were not willing to take SP (21%, 51/238).

### Suggestions for administration of SP for IPTi

Respondents at community and health facilities proposed that like IPT in pregnancy, IPTi should be delivered with direct observation by health workers. Otherwise, it was suspected that some mothers might throw SP away if they were asked to administer at home.

*Babies should be given that tablet [IPTi] right there [at clinic]; if we [mothers] take it out of the clinic, they [some people] may mislead us that the drugs are harmful so that we can throw them away *(FGD, mothers, village centre).

### Understanding existing posters at health facilities

Posters promoting vaccinations, malaria treatment and IPTp were displayed at all health facilities and some village offices. Some of these posters were misinterpreted and participants could often not recall their contents. For example, a poster with instructions about malaria treatment with SP was said to be too wordy. Another poster showing a child protected from six immunisable diseases (*TB, tetanus*, *polio, pertussis, diphtheria and measles*), represented by arrows and a shield, was understood by only a few respondents to show the importance of immunising children against the six diseases. It was explained by some Community Own Resource Persons that a shield was not known to a young generation in the study districts especially women. Hence, the intended meaning of protection might not always be understood. Much more frequently, respondents thought the poster showed that the child had all six vaccine-preventable diseases.

*"Those arrows suggest that the baby is being attacked by all those diseases"*. (FGD, mothers, village centre)

A second poster showing two pregnant women holding SP tablets for IPTp in the palms of their hands was well understood by some respondents to mean two doses of SP at different stages of pregnancy. Nevertheless, others were not able to tell what was shown on those posters, or had different interpretations.

*"I see two pregnant women with their mouths open; I don't know what else they are doing" *(FGD, mothers, outlying hamlet)

*"I see two girls who are in a bad condition, stretching their arms up as if to seek help" *(FGD mothers, outlying hamlet)

### Willingness to accept IPTi

Having heard about the results of the IPTi study in Ifakara [1], many respondents at health facility and community levels expressed their willingness to accept IPTi with the hope that it would reduce the nuisance of malaria, particularly sleepless nights (FGD, Distant Hamlet). Others thought that IPTi complements vaccines in children.

*"Vaccines are already available for many diseases such as measles but there is no prevention for malaria and it is a very big problem" (*Community Own Resource Person)

Some participants simply expressed their trust in IPTi if it was going to be delivered through routine health services, in close collaboration with researchers. There were a few reservations that it might be compromised by a shortage of health staff. Many respondents wanted information about the aim of the new intervention, to know what it consisted of, and to be reassured that it was safe.

### Communication channels for vaccination services

Health workers were mentioned in most sessions as the main source of information about vaccination and other child care services. Radio, community meetings at village and ward levels, announcements in churches and mosques, using a village crier and posters placed at health facilities, markets and big trees by the roadside were also mentioned, especially during mass immunisation campaigns. However, there were widespread complaints among mothers about infrequent health education sessions and inadequate explanations of the purpose of EPI vaccinations. On the other hand, some nurses alluded to mothers being uncooperative which barred their efforts to give health education.

*"When [we] educate them at clinic, they boycott. If you ask [a mother] which diseases are prevented by this injection, she tells you she doesn't know. A mother can say I don't know even if she is attending with her third baby" *(Public health nurse, district hospital).

### Development of BCC strategy for IPTi

Many respondents suggested that messages about IPTi should be delivered using the same channels as for routine vaccinations and during campaigns. Guided by the need for large-scale feasibility and low cost, the strategy focussed on interpersonal communication between health workers and mothers, supported by a brand name and posters.

### Brand name

FGD participants, health staff, and stakeholders at all levels were asked to suggest suitable brand names for IPTi. The aim was to find a culturally compelling and simple Swahili word or phrase containing concepts about prevention of malaria in infants linked to vaccinations. Many brands were suggested (Table 1) but were not adopted either because of possible negative connotations, no direct association with malaria prevention, or no link to vaccination. For example, the brand SHOKA (axe) was suggested as IPTi would 'chop down malaria as an axe chops down trees', but an axe is a potentially harmful tool and, therefore, not appealing as part of a brand name. OKOA (save) was suggested as this might convince mothers that the intervention would save lives, but there was no easy way to link OKOA with either malaria or EPI. The brand name MKINGE (protect him or her) was chosen because as this draws from a word that mothers often use for vaccines (*kinga*, meaning protect). When used as part of the phrase *"MKINGE mtoto wako dhidi ya malaria" *(protect your child from malaria) the connection between IPTi, malaria prevention and routine vaccinations is clear. Both training materials for health workers and the posters developed made extensive use of the "MKINGE" brand name for IPTi.

**Table 1:** Suggested brand names for IPTi

**Suggested brand name**	**Literal translation**	**Intended message**	**Remarks**
*SHOKA*	*An axe*	*IPTi will chop down malaria as an axe does for trees*	*Could be perceived as a harmful tool*
*MKIWA*	*None*	*Mpango wa Kinga ya Watoto i.e. a strategy for protecting children*	*MKIWA in Swahili language also stands for an orphan or hopeless person*
*LENGA*	*Target/shoot/aim at*	*IPTi targets malaria, anaemia and other child problems*	*There is no direct malaria-EPI and child link*
*OKOA*	*Save*	*To convince the mothers that this strategy saves lives*	*No direct malaria-EPI and child link*
*SHAMIRI*	*Flourish*	*Children will grow well and happily without diseases*	*No direct malaria-EPI link*
*MWAMBA*	*A rock*	*IPTi sounds like a rock where children can hide from malaria*	*No direct malaria-EPI link*
*NGUZO*	*A pillar*	*If a child leans on this strategy he/she will not fall down because of malaria and anaemia.*	*No direct malaria-EPI link*
*MKINGE*	*Protect him/her*	*Brand name MKINGE draws from the word kinga that mothers often use for vaccines.*	*When part of the phrase "MKINGE mtoto wako dhidi ya malaria" *(prevent your child from malaria), *MKINGE gives a positive image for IPTi linked to both malaria prevention and routine vaccinations*

### Posters

Building on the strengths and gaps on how existing posters were understood and suggestions from respondents, two posters for display at health facilities were developed and pre-tested. It was argued in various FGD sessions that mothers would get the messages from posters if they contained attractive images and clear messages. Illiterate mothers were said to ask others if they wanted to understand the written messages on health posters displayed at health facilities.

The posters were pretested in parts of Dar es Salaam, Lindi and Mtwara regions, to ascertain the clarity and of captions, images and design. Community representatives, health workers and influential people at district and national levels were also consulted to ensure the posters were technically sound and culturally appropriate.

#### Poster I: What is IPTi?

**Figure 1:**
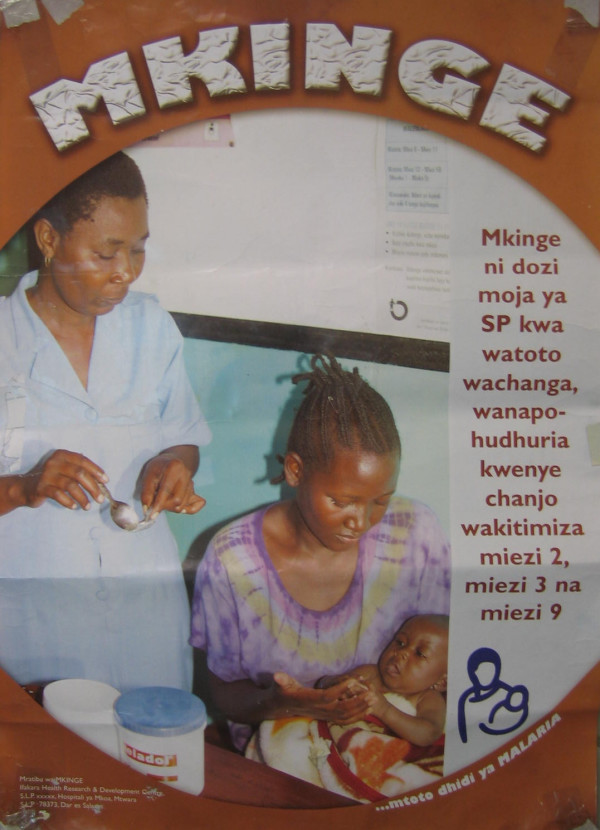
Draft version of poster to help health facility staff explain to mothers what IPTi is and when it is given.

The aim of this poster was to help health facility staff explain to mothers what IPTi is and when it is given. When pretested (Figure 1), the poster was perceived to show a single mother, whose dress make her look unhealthy, holding a baby, and an anxious nurse preparing medication ready for administering to the child. The caption "*Mkinge mtoto wako dhidi ya malaria" i.e*. "protect your child against malaria" was perceived to be too scattered. The background and font colours were not appealing to most respondents.

**Figure 2:**
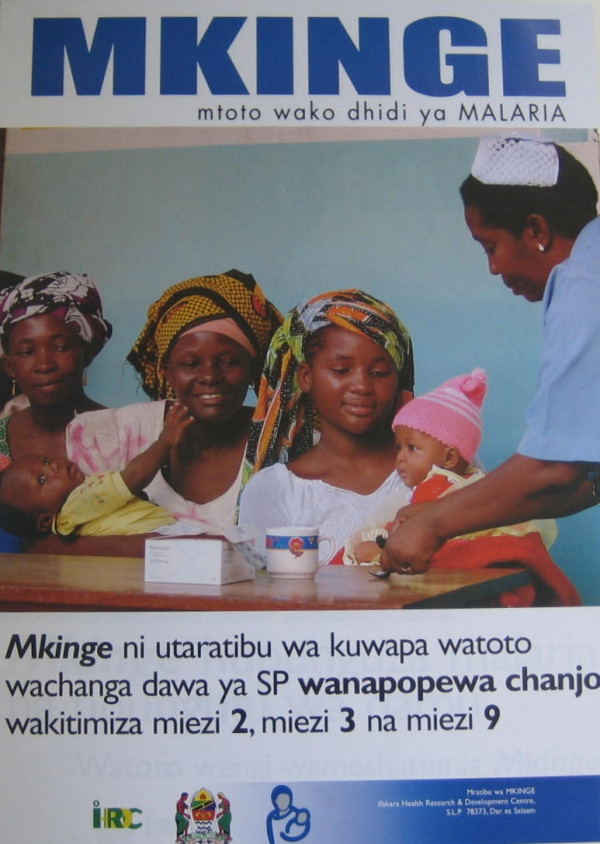
Final version of poster to help health facility staff explain to mothers what IPTis and when it is given.

The final version (Figure 2) was perceived in pretesting to be attractive, showing a friendly nurse giving IPTi to a baby, as other happy women with their babies queue for IPTi at a vaccination clinic. The captions read (1) "Protect your child against malaria" (2) "MKINGE is the delivery of SP to babies when they are given vaccines, at the age of 2, 3 and 9 months".

#### Poster 2: What does IPTi do?

**Figure 3:**
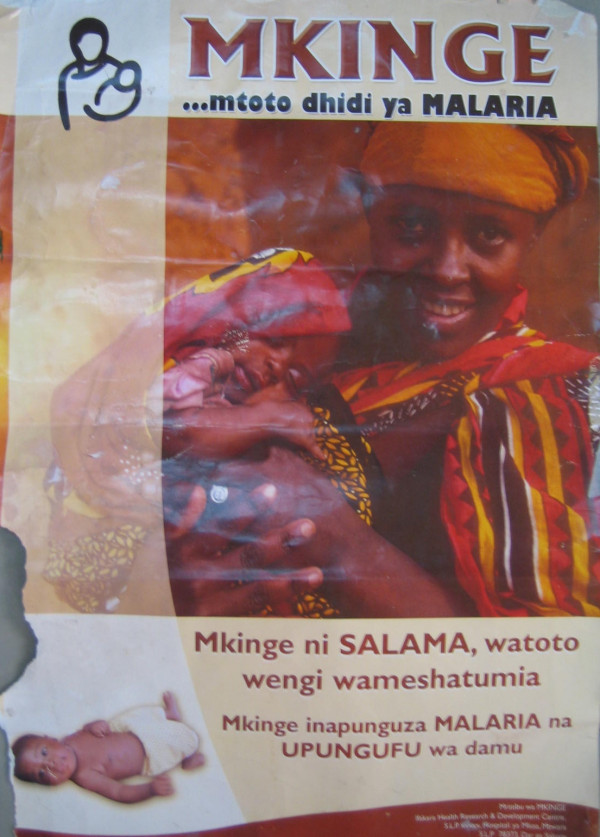
Draft version of poster to explain the benefits of IPTi to mothers and to reassure them about the safety of the intervention.

The aim of this poster was to explain the benefits of IPTi to mothers and to reassure them about the safety of the intervention. During pre-testing of the first draft (Figure 3), participants thought the sleeping baby was either dead or very sick. Another baby lying on the mat at the bottom left of this poster was also thought to be unhealthy.

**Figure 4:**
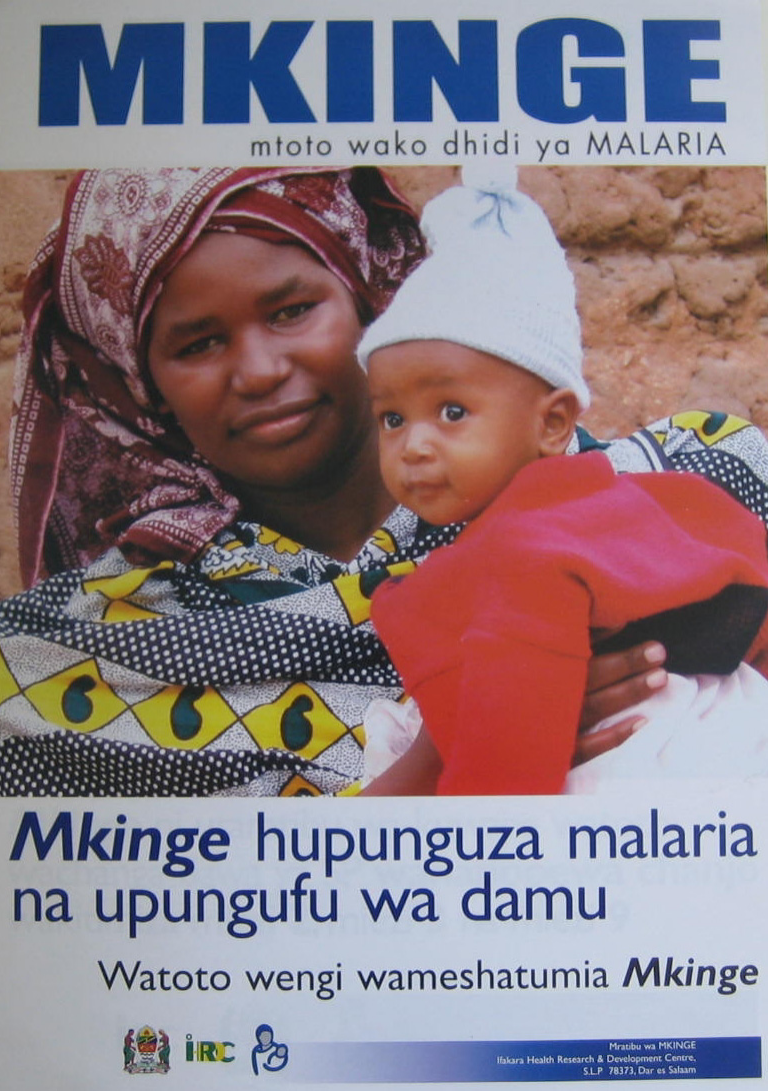
Final version of poster to explain the benefits of IPTi to mothers and to reassure them about the safety of the intervention.

The final version (Figure 4) shows a healthy mother and her child in a rural setting. There are three captions (1) "Protect your child from malaria" (2) "MKINGE reduces malaria and anaemia" (3) "Many children have already used MKINGE".

The posters also show the logo for the IPTi Consortium, the Tanzanian Ministry of Health and Ifakara Health Research and Developlement Centre (renamed Ifakara Health Institute in July 2008). The posters were produced and distributed to health facilities in the first quarter of 2005 to support implementation of IPTi.

## Discussion

The success of interventions and control programmes is moderated by local priorities and conditions, and the development of effective information, education and communication to support behavioural interventions requires good community based data [10]. Mixed research methods helped the study team to understand the local socio-cultural context about malaria, anaemia, SP and vaccinations before introduction of IPTi. The resulting local knowledge, experiences and expectations from community and health providers were used to inform the development of a BCC strategy for implementation of a new vaccination-linked malaria control tool in southern Tanzania.

Several women were interviewed who had probably used IPT in pregnancy but were unaware of it. There was no local term for IPT in pregnancy, and this could partly be due to lack of a brand name. A brand name helps to strengthen community knowledge and understanding of a new intervention, makes it easier to train health staff, and to manage, monitor and evaluate.

The success of health interventions depends upon an approach that is gender-sensitive [11]. Our findings suggested mothers were culturally accepted and expected to be key decision-makers for matters related to vaccination services. Mothers in our study took their babies for vaccination despite complaints of fevers and swellings after injections. In other settings, fear of side effects has been reported to deter attendance to EPI clinic [12]. Our findings suggest that concerns about side effects were outweighed by perceived benefits of weight measurement and vaccination together with avoiding the denial of treatment if the child's health card is incomplete.

There was an interest to explore rumours about SP because negative perceptions have been reported to affected behaviour and uptake of other reproductive health services [13]. Rumours about adverse effects of SP in our study featured in a few peri-urban settings, originating from hearsay and newspapers. Debates among researchers and decision makers prior to change of first line antimalarial treatment from chloroquine to SP also contributed to these widespread rumours [14]. Yet, many respondents had personally or knew others who safely used SP for malaria treatment or IPT in pregnancy. None of the respondents in our study had first-hand experience of adverse effects of SP and there were no vivid local examples about it. Implementation of IPTi with SP was, therefore, widely welcomed, especially after information that it would be through the routine health delivery system which they trusted. The extent to which the source of IPTi is trusted might influence decisions about IPTi.

### Study limitation

It should be noted that the data collection process was relatively rapid and might not facilitate in-depth understanding of socio-cultural issues. The findings were urgently required to inform development the BCC strategy.

### Advantages of approach used

Quantitative data on the coverage of EPI guided purposeful selection of the areas where qualitative data was collected. As an interactive process, the rapid qualitative data collection and quantitative health facility survey enabled involvement of local communities, front line health workers, district and national stakeholders in the development of BCC materials for IPTi.

Through this process, awareness was created and co-ownership of IPTi promoted in line with project's guiding principle of "together we develop IPTi". The methods used did not only help to inform development and pre-testing of BCC strategy but also helped in understanding of preferred communication channels from the viewpoint of the target audiences. FGDs general reveal "normal" behaviour and what is expected in a given society as people of similar characteristics freely interact to discuss pertinent issues in their real life context.

In deciding the BCC strategy, not all of the communication channels proposed were adopted – such as radio, ward and village meetings and using village criers. The meetings would be prohibitively expensive for large-scale implementation by the Ministry of Health. Radio would have led to information about IPTi reaching listeners living outside the project area. Instead, interpersonal communication between health staff and mothers was used, supported by posters and a brand name, images and messages that were prioritised, valued and resonant with the local culture, to enable a culturally compelling BCC strategy [15]. This was in keeping with approaches used by routine health services in sensitising communities about EPI and malaria interventions. Health workers were expected to use these posters in educating mothers about IPTi. This approach made implementation feasible and sustainable for use on a larger public health scale if and when needed.

## Conclusion

This study describes the development of a behaviour change communication strategy to support implementation of IPTi in southern Tanzania. This paper shows how mixed research methods were applied to inform the development and pretesting of BCC materials in view of local knowledge, experiences and expectations of community members and health providers as well as the Ministry of Health. The process used was intended to make the BCC materials culturally appropriate and technically sound. The BCC materials developed were made available in line with implementation phases of IPTi and are being evaluated in the context of monitoring the acceptability of this new malaria control tool.

## Competing interests

The authors declare that they have no competing interests.

## Authors' contributions

AKM participated in the study design, drafting and refining data collection tools, data collection and analysis and first draft of this manuscript. JAS, DS and RP provided technical input on the study design, data collection tools and data collection, and contributed to analysis and writing this manuscript. HM participated in designing and collection of the rapid qualitative study and commented on the manuscript, MM participated in designing and collection of rapid qualitative study and in Health facility Survey, CM and FM participated in designing the tools for Health Facility Survey and led data collection, HM, MT and PA provided technical input to the design of the study. All authors read and approved the final manuscript.
